# Mr. Bellingham on Hepatic and Pneumonic Inflammation

**Published:** 1813-09

**Authors:** R. Bellingham

**Affiliations:** Cuckfield, Sussex


*To the Editors of the Medical and Physical Journal.*

GENTLEMEN,

I OFFER the following case, merely to add one more instance to those already on record, of the ambiguity of the most prominent symptoms of pulmonic and hepatic inflammation; and of the probability of forming, without the closest attention, an incorrect diagnosis between them.

June 23d, I visited Mrs. Hartfield, a woman about 40 years of age. She complained of most acute pain of the chest; referred to a part just below the left breast; it was felt especially during inspiration, and was much aggravated by a recumbent posture. There was much oppression about the præcordia, with a frequent dry and painful cough. She had been laboring under these symptoms, gradually increasing, for three days, and the attack had been ushered in by repeated rigors; the pulse was quick and thready, about 110; the tongue much furred; the skin hot and dry; and the bowels torpid; she had constant nausea, and occasional vomiting. I immediately took ℥xvj of blood in a full stream

---

from from the arm, and ordered her the following pill at bed-time: ℞. Opii gr. j. Hydr. Submur. gr. iv. M. and in the morning a mixture containing Magnes. Sulph. ℥j.

24th.—The sickness was relieved, and the bowels briskly moved by the medicine. She had passed a quieter night; the pulse was rather more full, but the symptoms in other respects the same; ℥x of blood was taken from the arm, (that which I had previously taken exhibiting the strongest marks of inflammation;) a saline mixture directed to be taken every four hours, with an opiate at night.

25th.—The pain of the chest and cough still more severe; the mouth parched; the skin hot and dry. Repeated the blood-letting to the same extent; ordered a blister to the left side, and the following pills: ℞. Hydr. Subm. gr. iij. Ext. Colocynth. C. gr. xv. M. ft. pil. iv. statim sumendæ. The saline medicine was continued, with the addition of Tinct. Digital. gtt. v. to each dose, and she was desired to drink Infusum Lini, with a small quantity of nitre in solution.

26th.—She considered herself much worse; had passed a very restless night; countenance was expressive of the greatest anguish; tongue, teeth, and lips, covered with dark sordes; the pulse 126. The pills had produced several dark-colored and very offensive evacuations. The little relief she had hitherto experienced from general blood-letting, together with the appearance of the alvine discharge, made me doubtful, notwithstanding the reference of the patient's feelings, whether the thoracic viscera were really the seat of inflammation; and upon examining with greater care than I had previously done, I found very considerable tenderness on pressure about the region of the liver, and, as far as I could judge by the most cautious touch, an unusual degree of fulness. I immediately took away a few ounces of blood, by means of cupping glasses applied to the margin of the ribs, and ordered her the following pill to be taken every four hours. ℞. Hydr. Subm. gr. j. Ext. Colocynth. C. gr. v. M.

27th.—The acute pain of the chest had entirely left her, and she now complained of pain, of the obtuse kind, of the right side. The tunica albuginea of the eyes was becoming slightly suffused with yellow; the urine high-colored. Leeches were applied to the hypochondrium, and drew blood freely. The pills and opiate at night were continued.

28th.—The tenderness of the side lessened; cough very troublesome. The whole skin was now becoming strongly tinged with bile. The pills had produced frequent evacuations, the appearance of which improved. Applicetur Hypochondrio dextro Emplast. Lyttæ Amplum.

29th.—Feels much easier. Pulse 130 and very small. Had been for a short time in a general perspiration. ℞. Hydr. Submur.

Submur. gr. i. Opii, gr. ¼. ft. pilula 6ta quaque horâ sumenda. ℞. Infus. Calumbæ ℥vj. Bibat 4tam partem 4ta quaque horâ.

She continued these remedies for a few days, occasionally interposing a dose of the Ext. Colocynth. C. with the most manifest advantage. The evacuations from the bowels were much better, and it became evident that the liver was resuming its healthy function. The ictoritious color of the skin soon disappeared.

July 3d.—Apthous ulcerations of the mouth and throat troubled her so much as almost to prevent her swallowing; and she had at the same time severe griping and irritation of the bowels. These were soon relieved, the former by the use of astringent gargles, the latter by opiates. Scaly eruptions appeared afterwards about the mouth and nostrils. From this time she was quite convalescent. She necessarily remained in a state of great debility, but from this, by the assistance of tonic medicines and a more generous diet, she is now fast recovering.

That this was a genuine case of hepatitis, few, I apprehend, with even this imperfect detail before them, will be more disposed to doubt than myself. It shows how easily we may be misled if we are guided only by the reference of a patient's feelings, and how necessary it is to trace the seat of disease by manual examination, which in a case like this will be an unerring guide.

The complaint had proceeded to its greatest height before the least degree of pain was felt in the region of the liver, and the transition afterwards was remarkable. The acute pain of the left breast ceased entirely from the moment that of the right side was first complained of, nor was this in the least degree owing to the patient's attention being diverted from the former.

That general blood-letting in inflammation of this viscus should give *less*, while local bleeding and purging should afford *greater* relief than in pneumonia might, *à priori*, be expected, when we take into account the peculiarity of the hepatic circulation, the disproportionate quantity of arterial to that of venous blood it transmits, and the office it performs.

I will not occupy your Journal with an useless speculation upon the particular *modus operandi* of local bleeding in this disease. The plain fact few practitioners will deny, though each, perhaps, may suggest a different theory to explain it. That it proved in this case, and has proved in many others of a similar kind, far more useful than the abstraction of any quantity of blood from the arm, I have not the smallest doubt. I have the honor to be, Gentlemen,

*Cuckfield, Sussex,* Your most obedient Servant,

*July* 18, 1813. R. BELLINGHAM.

